# What Does It Mean to Successfully Age?: Multinational Study of Older Adults’ Perceptions

**DOI:** 10.1093/geront/gnae102

**Published:** 2024-08-10

**Authors:** Elissa Burton, Barbra Teater, Jill Chonody, Sabretta Alford

**Affiliations:** Faculty of Health Sciences, enAble Institute, Curtin University, Perth, Western Australia, Australia; Faculty of Health Sciences, Curtin School of Allied Health, Curtin University, Perth, Western Australia, Australia; Department of Social Work, College of Staten Island, City University of New York, Staten Island, New York, USA; School of Social Work, College of Health Sciences, Boise State University, Boise, Idaho, USA; The Graduate Center, City University of New York, New York, New York, USA

**Keywords:** Multicountry, Older adults, Successful aging

## Abstract

**Background and Objectives:**

Successful aging is a mainstay of the gerontological literature, but it is not without criticism, including the often-limited way that it is studied and measured as well as the exclusion of older adults’ voices in its formulation and understanding. This study sought to address these issues through a qualitative investigation across multiple countries.

**Research Design and Methods:**

This was a mixed-methods, cross-sectional, exploratory study using an online survey. Nations that received the survey included Australia, New Zealand, the United Kingdom, Ireland, Canada, and the Unites States. Participants aged 65 and older were asked to describe what successful aging means to them in an open-ended survey item. Summative content analysis was utilized to examine the responses.

**Results:**

Successful aging was defined by 1,994 participants, and 6 themes along with 20 subthemes were found. In contrast to conception that successful aging is solely or predominantly related to the absence of disease and decline, the most prominent theme in this study was “active, independent, and engaged” as the hallmark of success.

**Discussion and Implications:**

Although health and health maintenance were present in other themes, these findings support a multidimensional definition of successful aging that promotes the perspectives of older people. Future research should seek to further investigate the ways in which person-in-environment factors influence definitions of successful aging, including culture, gender and gender identity, race and ethnicity, and socioeconomic background.

Successful aging is a prominent gerontological paradigm particularly in Western countries, and since its introduction, it has shaped aging perceptions, particularly in the United States. Originally discussed in the substantive literature by Havighurst (1961), who argued a theory of successful aging should include elements of satisfaction and happiness (Martin et al., 2015), the contemporary version of successful aging is derived from Rowe and Kahn’s (1987, 1997, 1998) framework, which bifurcated aging into those who aged “usually” and those who aged “successfully.” These successfully aging individuals had lower health risks and greater functioning, which includes a combination of physical, cognitive, and lifestyle factors. In particular, Rowe and Kahn (1997) theorized successful aging as: (a) low probability of disease and disease-related disability; (b) high cognitive and physical functional capacity; and (c) active engagement in life. Successful aging was achieved through healthy maintenance and “full engagement in life, including productive activities and interpersonal relations” (Rowe, 1997, p. 367); thus, lifestyle choices that maintain health and reverse disabling problems are emphasized. Arguably, this “move(s) successful aging further from the social determinants of health” (Katz & Calasanti, 2015, p. 27) and away from an acknowledgment of the influence of the environment on the aging process (Teater & Chonody, 2017).

The term “successful aging” is ubiquitous in gerontological literature and frequently used interchangeably with active aging, healthy aging, optimal aging, and productive aging (Teater & Chonody, 2020b). Despite expansions on the definition of successful aging by other theorists and researchers (see Estebsari et al., 2020; Martin et al., 2015; Michel & Sadana, 2017 for a review of these definitions), attempts to identify and measure successful aging have predominantly focused on one or more of the three components as identified by Rowe and Kahn (1997), thus leaving a limited and unclear picture of the extent to which people are aging successfully (Teater & Chonody, 2020b). Moreover, rigid retention to Rowe and Kahn’s (1997) three criteria excludes many older adults, in particular those who live with dependency and disabilities (Carver & Buchanan, 2016; Martinson & Berridge, 2015; Teater & Chonody, 2017), and represents an ageist bias in that older people are expected to maintain middle-age standards (Calasanti, 2003). Katz and Calasanti (2015) argue if older adults are “considered unsuccessful agers in theory, then such labeling deeply affects their treatment by health care regimes in practice” (p. 29), which will be exacerbated through an intersectional lens of gender and gender identity, sexuality, race and ethnicity, and socioeconomic status (Teater & Chonody, 2020b).

A unified definition and one that extends beyond Rowe and Kahn’s (1997) framework (Katz & Calasanti, 2015; Teater & Chonody, 2020b) is necessary for inclusive research efforts. The largely missing perspectives of older adults (Cosco et al., 2013; Teater & Chonody, 2020a) in the development of such a definition and the overreliance on biomedical constructs of this concept (Carver & Buchanan, 2016) are also fundamental criticisms of the existing literature. As a result, researchers have attempted to capture the views of older people through qualitative methods (see Cosco et al., 2013; Teater & Chonody, 2020a for a review of the literature) or via systematic reviews of the literature that examine the definitions of successful aging and the themes that emerge across definitions (Bowling & Dieppe, 2005; Depp & Jeste, 2006; Howell & Peterson, 2020; Reichstadt et al., 2010; Teater & Chonody, 2020c). Although research is expanding the knowledge base and definition of successful aging, existing studies are often focused on one geographical area or are with small sample sizes. Thus, there is also a dearth of literature conducted across countries allowing for direct comparison of cultures and environments (Reich et al., 2020).

The existing research on successful aging indicates that aging adults do not consistently view disease and disability as unsuccessful and have identified other factors that influence their ability to age successfully (Teater & Chonody, 2020b). Perspectives on successful aging from older adults have illustrated the multidimensional nature of this concept that includes both internal (bio-psycho-social-spiritual) and external (socio-political-economic) factors (Teater & Chonody, 2020b). For example, financial capability and access to healthcare (external factors) as well as psychological elements, including acceptance, coping, self-determination, and resilience (internal factors) were identified in the responses to “what does successful aging mean to you?” (Howell & Peterson, 2020; Teater & Chonody, 2020b, 2020c). Additionally, other factors such as spirituality and/or religiosity and gerotranscendence (i.e., the natural progression toward wisdom and maturation) have emerged as internal facets of successful aging (Howell & Peterson, 2020; Teater & Chonody, 2020b, 2020c). Capturing this rich description can help move the discourse of successful aging away from individual choice and responsibility to a more comprehensive view of the person in the environment (Teater & Chonody, 2020b).

What it means to “age” and what is needed to create an environment to “age successfully” are narratives that often influence policies and practices towards older adults. How older adults view successful aging can shed light on the issues that are important to them and may assist in improving service delivery practices and policies for older people. This study sought to answer the following research questions:

1. Do older adults think they are aging successfully?
2. How do older adults define successful aging, and what components comprise such definitions?

## Method

### Design

This was a mixed-methods, cross-sectional, exploratory study, distributed by online survey using Qualtrics from September 2021 to April 2022.

### Setting and Sample

The survey was sent out to older adults (aged 65 years and older) predominantly via Facebook advertisements targeting people living in Australia, New Zealand, the United Kingdom, Ireland, Canada, and the United States. It was also disseminated to participants on a research database (*n *= 150), a link on the Council on the Aging Western Australia newsletter, the Strength for Life newsletter, and the Injury Matters e-newsletter. These newsletters were used to provide some diversity outside of Facebook users.

Inclusion criteria included being aged 65 years or older (self-reported), understanding and able to complete the survey in English, and access to the internet and/or social media. If a person did not meet these criteria, they were unable to access the survey. Prior to the survey being distributed, it was pilot tested with four older adults (two from regional Australia, one from metropolitan area Australia, and one from Scotland) and one minor change was made (i.e., asking for age rather than birth year). Participants were not compensated for completing the survey. Ethical approval was received from the Curtin University Human Research Ethics Committee (HRE 2021-0587) and each participant provided consent prior to accessing the online survey.

### Instrumentation and Data Collection

Participants completed demographic questions about themselves, including age, sex, where and who they lived with, level of education, employment status, children, medications, and activities of daily living. They were also asked to answer the following questions: “Please describe what ‘successful aging’ means to you?” (open-ended question) and “Do you feel like you are successfully aging?” (1 = *definitely not* to 5 = *definitely yes*). When asking the question “Please describe what ‘successful aging’ means to you?” no examples were provided, limiting bias of the researchers to influence the participants’ answers through the use of check boxes or examples from earlier research.

Four thousand, two hundred and forty-seven participants clicked on the survey link and 2,975 provided consent and met the inclusion criteria. Of these, 1,994 (67%) survey respondents answered both questions described above and the majority of participant demographic questions to be included in the study.

### Data Analysis

IBM SPSS Statistics (version 27, Armonk, NY) was used to analyze the quantitative data including participant demographics and research question one. Continuous data were checked for normality. Data were summarized using means and standard deviations for continuous data and frequency distribution for categorical data or nonparametric tests were used as required. *T* tests, analysis of variance (ANOVAs), and chi-square tests were used for comparisons. Significance levels were set at .05.

The qualitative data analysis followed a summative content analysis approach (Hsieh & Shannon, 2005) where each participant response was read and then counted and collated with like responses from other participants. The existing words or phrases within each participants’ responses were examined for latent meanings and themes across the responses. The summative content analysis consisted of the following steps, as detailed by Lune and Berg (2017). First, the data were transferred into an Excel spreadsheet to be organized to read. Next, each response was read and reread to generate initial codes of one word or a short phrase that encapsulated the meaning of the data extracted by one researcher. Each participant’s response generated between 1 and 12 codes resulting in 4,246 initial codes. Examples of initial codes included: engaged in society; not lonely; new experiences; wisdom; and balance. Third, the initial codes recorded on an Excel spreadsheet by each participant response were reviewed and discussed by all four researchers, and one researcher then combined all duplicate codes and collated similar codes. This process resulted in 238 codes. Fourth, the 238 codes were further reviewed and refined into categorical labels or themes, which resulted in 22 themes. Examples of themes included: social connection; capabilities; acceptance; spirituality; and physical well-being. Fifth, the themes were reviewed and discussed by all researchers and sorted by rereading the data under each theme to ensure they formed a coherent pattern. Themes were refined by collating similar themes and moving themes into subthemes of a larger overarching theme. For example, themes of “social connection” and “support” were combined into a theme of “social-emotional connection and support,” and themes such as “future planning” and “hobbies” were moved to subthemes of “actively engaged and independent.” Sixth, the overarching themes were then reviewed and discussed by all researchers and refined to tell an overall story of what constitutes successful aging. This resulted in six overarching themes and 20 subthemes. Lastly, the findings are presented below by describing each theme followed by a discussion of the findings considering previous research and theories.

#### Trustworthiness

The following actions were taken to ensure the trustworthiness of the data analysis and presentation of the findings. First, three of the four coders were highly experienced in qualitative research having published >62 qualitative articles combined. The fourth coder was mentored closely throughout the process to assist them in becoming a proficient qualitative researcher (they currently have one published mixed-methods paper). Second, dependability was enhanced by the authors creating an audit trail of the data analysis to detail how the participants’ responses were analyzed into six themes and 20 subthemes. Third, credibility was enhanced by providing direct quotes from the participants to support each theme and subtheme in order to retain participants’ original expression of their views on successful aging. Finally, credibility was further enhanced by all four authors reviewing the codes and themes individually and in regular peer debriefing sessions to have a consensus on the final analysis (Lincoln & Guba, 1985).

## Results

### Sample Demographics

The average age of the participants was 72.7 (± 5.8) years with a range of 65 to 99 years. Three quarters of the sample were female, 35% were born in the United Kingdom; however, 35% were living in Australia when they completed the survey (see Table 1). There was a reasonably even mix between areas respondents lived in between regional area (i.e., small town), regional city, and capital city or surrounding suburbs. This was a predominantly well-functioning group with the majority (96%) completing activities of daily living with no difficulty.

**Table 1.** Sample Demographics

Variables	Value	*N* with data
Age Mean (*SD*)	72.73 (±5.76)	1,985
Range	65–99	
Median (IQR)	72 (68, 76)	
Sex % (*n*)		1,994
Female	74.7 (1,490)	
Male	24.0 (479)	
Non-binary	0.4 (7)	
Transgender	0.1 (2)	
Intersex	0.1 (2)	
I prefer not to say	0.7 (14)	
Country born in % (*n*)		1,980
United Kingdom	34.9 (691)	
Australia	18.6 (369)	
Canada	16.7 (330)	
United States of America	9.3 (185)	
New Zealand	6.5 (129)	
Ireland	6.3 (125)	
Netherlands	1.2 (23)	
Germany	0.6 (12)	
Other	5.9 (116)	
What region do you live in % (*n*)		1,994
Australasia (Australia and New Zealand)	35.5 (683)	
United Kingdom-Ireland-Europe	28.7 (552)	
North America (Canada and United States)	28.0 (538)	
Other not specified	7.7 (149)	
Other specified	0.1 (2)	
Country live in currently % (*n*)		1,916
Australia	28.3 (544)	
United Kingdom	20.9 (403)	
Canada	20.4 (392)	
United States of America	7.6 (146)	
Ireland	7.5 (144)	
New Zealand	7.2 (139)	
Other	8.1 (156)	
Area you live in % (*n*)		1,848
Capital city/suburbs surrounding	26.9 (497)	
Regional city	29.9 (553)	
Regional area (e.g., small town, farming)	39.0 (720)	
Remote	4.2 (78)	
Highest level of education % (*n*)		1,861
Primary school	1.9 (35)	
High school	24.3 (453)	
Trade or apprenticeship (non-university training)	21.0 (390)	
Undergraduate degree	26.8 (500)	
Postgraduate degree	26.0 (483)	
Employment status % (*n*)		1,865
Retired	82.1 (1,532)	
Working part-time	12.2 (227)	
Working full-time	5.1 (95)	
Unemployed	0.6 (11)	
Living status % (*n*)		1,866
Live alone	33.3 (622)	
Live with spouse/partner	57.8 (1,078)	
Live with other family (or they live with you)	7.3 (137)	
Live with friends (or they live with you)	0.7 (13)	
Live with other people not mentioned above	0.9 (16)	
Number of children Mean (*SD*)	2.25 (± 1.33)	1,786
Range	0–9	
Prescribed medication taking Mean (*SD*)	3.12 (± 2.88)	1,773
Range	0–25	
Median (IQR)	2 (1, 4)	
Able to complete activities of daily living		1,870
Complete without difficulty	96.0 (1,795)	
Some difficulty, need a little help	3.4 (63)	
Moderate difficulty and need help	0.5 (10)	
Significant difficulty needs quite a bit of help	0.1 (2)	
Help in the home (paid or family) % (*n*)		1,869
Yes	20.2 (378)	
No	79.8 (1,491)	

*Note*: IQR = interquartile range.

Participants predominately reported living in three world regions (i.e., United Kingdom-Ireland, Australia-New Zealand, and Canada-United States). An additional group called “other” was created for those surveys with missing data for their country. Participants from the United Kingdom and Ireland were significantly younger than Canada and the United States (see Table 2). Significantly more Australians and New Zealanders lived in capital cities than the other regions and significantly more from the United Kingdom and Ireland lived in regional areas and fewer in regional cities. Australia and New Zealand had significantly more participants with a postgraduate degree than those from the other two regions. Australia and New Zealand respondents were also taking significantly more prescribed medications than the Canadian and American participants.

**Table 2.** Sample Demographics Per Region

Variables	Australia and New Zealand	United Kingdom and Ireland	United States and Canada	Other (not specified)
Number participants	676	546	533	144
Age *Mean* (*SD*)	72.75 (5.60)	71.47 (4.87)^**^	73.60 (6.28)	73.33 (6.00)
Range	65–99	65–89	65–95	65–95
Sex % (*n*)#				
Female	78.8 (533)	74.9 (409)	73.4 (391)	72.2 (104)
Male	21.2 (143)	25.1 (137)	26.6 (142)	27.8 (40)
Area you live in % (*n*)				
Capital city/suburbs surrounding	41.2 (274)^**^	14.5 (77)	24.1 (127)	14.6 (15)
Regional city	34.0 (226)	20.0 (106)^*^	33.4 (176)	36.9 (38)
Regional area (e.g., small town, farming)	23.6 (157)	59.7 (317)^*^	37.2 (196)	40.8 (42)
Remote	1.2 (8)	5.8 (31)	5.3 (28)	7.8 (8)
Highest level of education % (*n*)				
Primary school	1.4 (9)	3.0 (16)	1.1 (6)	<5
High school	24.2 (161)	22.3 (121)	23.9 (126)	38.7 (41)^**^
Trade or apprenticeship (non-university training)	21.7 (144)	22.0 (119)	19.0 (100)	21.7 (23)
Undergraduate degree	22.7 (151)	28.8 (156)	31.1 (164)	20.8 (22)
Postgraduate degree	30.0 (199)^**^	24.0 (130)	24.9 (131)	15.1 (16)
Employment status % (*n*)				
Retired	80.2 (533)	83.6 (453)	82.1 (435)	87.7 (93)
Working part-time	12.9 (86)	12.2 (66)	12.5 (66)	6.6 (7)
Working full-time	6.0 (40)	4.1 (22)	4.7 (25)	5.7 (6)
Unemployed	0.9 (6)	<5	<5	<5
Living status % (*n*)				
Live alone	34.5 (232)	32.1 (176)	33.4 (179)	31.5 (35)
Live with spouse/partner	54.4 (366)	60.9 (333)	58.1 (311)	61.3 (68)
Live with others	11.1 (75)	6.9 (38)	8.4 (45)	7.2 (8)
Number of children *Mean* (*SD*)	2.35 (1.36)^*^	2.26 (1.33)	2.13 (1.25)	2.22 (1.35)
Prescribed medication taking	3.03 (2.84)	2.97 (3.00)	3.31 (2.87)	3.40 (2.76)
Able to complete activities of daily living				
Complete without difficulty	95.8 (638)	96.3 (526)	96.0 (510)	95.2 (100)
Some difficulty, need a little help	3.5 (23)	2.7 (15)	3.6 (19)	4.8 (5)
Moderate difficulty and need help	0.8 (5)	<5	<5	<5
Significant difficulty - needs quite a bit of help	<5	<5	<5	<5
Help in the home (paid or family) % (*n*)				
Yes	23.1 (154)	16.9 (92)	20.3 (108)	18.1 (19)
No	76.9 (512)	83.1 (452)	79.7 (424)	81.9 (86)

*Note*:

^*^ *p* < .05.

^**^ *p* < .001.

### Perceived to be Successfully Aging

Almost 85% of survey participants perceived they were probably (43.9%, *n* = 875) or definitely (41.0%, *n* = 817) successfully aging, as opposed to 6.4% that felt they were definitely not (1.9%, *n* = 38) or probably not (4.5%, *n* = 90) successfully aging, and 8.7% (*n* = 174) were unsure whether they were successfully aging. When comparing perceptions of successful aging per region, the only significant difference was that fewer participants in the United Kingdom and Ireland (33.9%) reported “definitely” feeling like they were successfully aging compared to those in Australia and New Zealand (45.3%). Table 3 reports on each region.

**Table 3.** Perceptions of Successfully Aging Per Region

Variables	Australia and New Zealand	United Kingdom and Ireland	United States and Canada	Other (not specified)
Do you feel like you are successfully aging? % (*n*)				
Definitely not	2.4 (16)	1.6 (9)	1.9 (10)	<5
Probably not	4.0 (27)	4.6 (25)	4.9 (26)	4.2 (6)
Unsure	6.7 (45)	9.2 (50)	10.9 (58)	7.6 (11)
Probably yes	41.7 (282)	50.7 (277)	39.4 (210)	46.5 (67)
Definitely yes	45.3 (306)	33.9 (185)^*^	43.0 (229)	41.0 (59)

*Note*:

^*^ *p* value = .005.

### Components of Successful Aging

**Figure 1.**
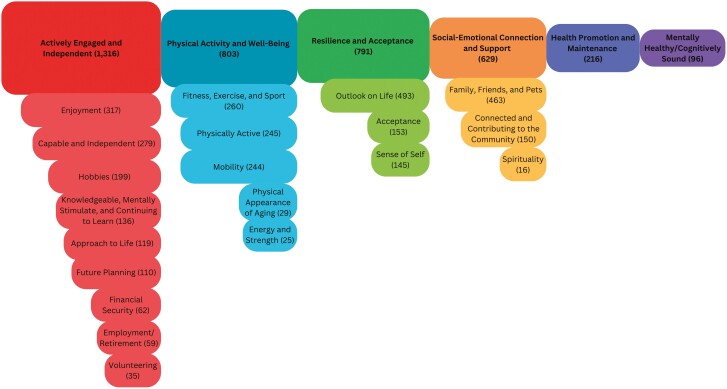
Themes and subthemes of successful aging.

Figure 1 depicts the six themes and 20 subthemes that summarize participants’ responses to: “Describe what successful aging means to you” along with the frequency in which the themes or subthemes were identified among the participants’ responses. Some of the quotations shown below may apply to more than one component. Six themes were found: Actively Engaged and Independent; Physical Activity and Well-being; Resilience and Acceptance; Social-Emotional Connection and Support; Health Promotion and Maintenance; and Mentally Healthy/Cognitively Sound. These themes illustrate the multidimensional aspect of successful aging among the participants, which was found both within participants’ single response to the question as well as the combination of responses among participants. For example, one participant provided the following description of successful aging:

Maintaining good health, controlling any chronic diseases. Having good energy levels and emotional and mental health under control. Having good support from close ones. A good social circle. Plenty of interests. The ability to relax and look after yourself whilst also considering those close to you. Financial security is also important. (#9)

Other participants responded with a single component when describing successful aging, such as: “being content with life” (#450); “healthy” (#1003); “being totally independent and compos mentis” (#1048); and “being happy and content” (#1107). Each of the six themes are described in detail below through the 20 subthemes with direct quotes provided by the participants.

#### Actively engaged and independent

Descriptions of successful aging were predominately related to aspects of being actively engaged in life and maintaining independence (*n* = 1,316), and the nine subthemes capture this: Enjoyment; Capable and Independent; Hobbies; Knowledgeable, Mentally Stimulated, and Continuing to Learn; Approach to Life; Future Planning; Financial Security; Employment/Retirement; and Volunteering. Enjoyment was important for many participants (*n* = 317) and included simply “enjoyment of life” (#310) and “enjoyment of whatever ‘floats your boat’” (#843), or more elaborated comments, such as “enjoyment, acceptance, modification, a reason to get up in the morning” (#210), “having more time to do things that bring enjoyment including doing nothing” (#388), and “being able to do the things I want to do and still be able to do them and get enjoyment from them” (#661). Capable and independent was mentioned by 279 participants, which included: “continue to do all the things you are capable of and adjust if you’re not” (#1165); “being capable of doing everything without help” (#1606); “being independent and able to live in your own home” (#1602); and “being able to get out of bed in the morning and being independent both physically and mentally” (#1683).

Participants also described the importance of engaging in hobbies (*n* = 199), which could mean starting a new hobby: “For me it means I made it this far and am looking forward to start to develop my hobbies” (#1880) or continuing to engage in hobbies, for example, “I hope to continue to pursue my interests and hobbies” (#1967). Other participants described successful aging as being knowledgeable, mentally stimulated, and continuing to learn (*n* = 136). These responses included: “maintaining an interest in continuous learning” (#1990), or “…learning something new every day” (#2225) or “keeping your brain active and stimulated” (#100). Some participants viewed successful aging as having a particular approach to life (*n* = 119), such as: “I suppose for me it’s accepting that whether I like it or not I am now of a certain age but at the same time retaining, hopefully, a broad-minded attitude and approach to life” (#1019) or to “approach every challenge with grace” (#4122).

Looking toward the future and engaging in future planning (*n* = 110) was also part of this theme. For example, one participant stated, “health adequate wealth and a plan for the future with a few challenges” (#2234). Others extended this future planning to include financial security (*n *= 62) as one participant described, “living a full and healthy life with the financial security to ensure access to participation in those activities which promote both physical and mental wellbeing” (#3621) while other participants merely mentioned “financial security” (#3347; 3960). Staying engaged in employment or enjoying retirement (*n* = 59) and volunteering (*n* = 35) were important to some participants when describing successful aging, which often overlapped with financial security: “able to be financially secure, traveling, volunteering, or having paid employment as desired” (#3949) and “coping with retirement: reduced income, focus and status” (#4238), and “living the years of retirement actively with personal fulfillment, service to others, and a minimum of regrets” (#4242).

#### Physical activity and well-being

Remaining physically active and having physical well-being was the second most prominent theme (*n* = 803) with subthemes of: Fitness, Exercise, and Sport; Physically Active; Mobility; Physical Appearance of Aging; and Energy and Strength. Remaining physically active through fitness, exercise, and sport (*n* = 260), for example, participants described, “maintaining good health and an appropriate level of fitness through a healthy lifestyle—i.e., diet, exercise and mental health” (#5); “managing health and fitness to sustain quality of life” (#27); and “being active, healthy, involved in life, family, sport, clubs, etc.” (#376). Other participants were less specific on fitness, exercise or sport, yet still expressed the need to be active (*n* = 245) or have mobility (*n* = 244) for successful aging. Some examples of their responses included: “having mobility, both physical mobility, and to have access to a car or other convenient transport” (#374); “staying fit and healthy, active and interested” (#403); “to be as physically active and alert as possible through healthy lifestyle” (#430); “mobility maintained at a good level” (#522); and “mobility and general well-being” (#804).

For a few participants, successful aging involved managing one’s physical appearance (*n* = 29) by “try[ing] to look you[r] best and take pride in your appearance” (#901); “…dress nicely, don’t look like a really old person” (#2413); or “looking younger than your age” (#3303). For other participants, successful aging meant having energy and strength (*n* = 25) such as “having good health with energy to take care of myself” (#4244); or “…having good energy levels” (#9).

#### Resilience and acceptance

Participants reported the importance of resilience and acceptance (*n* = 791) to successful aging. Subthemes that described resilience and acceptance included: Outlook on Life; Sense of Self; and Acceptance. Participants pointed to the importance of outlook on life (*n* = 493) for successful aging, which included: “having a positive outlook on life. Continuing to take a lively interest in the world around us. Maintaining a social circle. Not living in the past or dwelling on memories of times gone by” (#132); “being of sound mind, active, within realms, and having a positive outlook on life” (#719); or “being in good health and having a good outlook on life” (#1191). Other participants acknowledge an inward view as significant to successful aging, particularly one’s sense of self (*n* = 145) where successful aging “…starts by knowing thyself” (#3645); “…continuously discovering self” (#3900), which may be through “a sense of engagement and usefulness in social and community activities” (#283). Finally, some participants acknowledge an element of acceptance (*n* = 153) to successful aging. One participant describes:

Successful aging, from my perspective, is coming to terms (accepting and understanding) that there are lots of things I could once do that are now just memories and being OK with that and not beating myself up over not being the man I was. A large part of this acceptance/understanding paradigm is not seeing [it] as a loss, for there are benefits. (#320)

Other participants refer to acceptance as: “finding a level of contentment with life, being accepting of the good and the bad I’ve experienced throughout my life” (#817); “acceptance of the aging process” (#1221); and “staying well amidst suffering and radical acceptance of everything including how my body is now” (#1271).

#### Social-emotional connection and support

Participants described elements of social-emotional connection and support (*n* = 629) as important to successful aging with the following subthemes: Family, Friends, and Pets; Connected and Contributing to the Community; and Spirituality. Having family, friends, and pets (*n* = 463) was important for many participants when describing successful aging, which was often stated simply, such as: “have a good respectful and loving family and loyal friends and neighbors” (#1331); “having good friends and family” (#1362); “remaining a part of my family’s life” (#1389); “enjoying family and friends, including making new ones” (#1418); “…care for my dogs” (#1823); and “having a pet, especially if living alone” (#2178). Additionally, the family, friends, and pets were also viewed as providing support to the participants, such as: “having good support from close ones” (#9); “the company and support of friends and family” (#29); “having a good support network of family and friends” (#330); and “social contacts and the support of family” (#821).

Being connected and contributing to the community (*n* = 150) was also identified as important to some participants in creating and sustaining social-emotional connection and support. One participant described successful aging as “…giving back to the community in terms of voluntary work at the highest level of your skillset” (#336) and another participant stated, “continuing as I age, to be involved, engaged, contributing to my community and the world” (#3440). Other participants simply stated, “contributing to the community” (#2184; 2319). Finally, spirituality (*n* = 16) was important to a small number of participants who either merely listed “spirituality” (#4127) or included spirituality alongside other components, such as: “continuing to be healthy and active, physically, socially, mentally and spiritually” (#129). One participant indicated the importance of spirituality in making choices as they age:

To be free to make choices as to how I wish to live my life within the bounds of my moral and spiritual compass that I have maintained all my life even if I no longer have capacity and that others nominated to be able to enforce that are able to dictate that for me. (#94)

#### Health promotion and maintenance

Some participants mentioned the importance of maintaining their health, but also their role in preventing ailments and health problems, which was different from the theme of Physical Activity and Well-Being, which described engaging in activities that required mobility, energy, and strength. The theme of health promotion and maintenance (*n* = 216) included comments from participants who described successful aging as: “being as well as I can be as I get older. Looking after my body. Taking preventative measures for as long as I can” (#1114); having “preventative tests” (#4192); “attend[ing] to one’s health; having screening tests where necessary. Preferably having a regular GP and a good relationship with them” (#85); and “keeping healthy … by which I mean preventative maintenance … keeping up health checks” (#120).

#### Mentally healthy/cognitively sound

A small number of participants expressed being mentally healthy and cognitively sound (*n* = 96) as components of successful aging. For some participants, “mental acuity and stability” (#132) were mentioned in a list of other items, such as “staying fit and healthy physically and mentally” (#272); or “remaining physically and mentally active for as long as possible” (#297). Other participants placed a stronger emphasis on mental capacity, such as “…nor do I want to lose my mental acuity” (#322); “being mentally able to make decisions” (#199); “the mental acuity to derive pleasure from mentally challenging and stimulating activities” (#283); or “to be able to understand what’s going on around me and, in the world in general. Mental clarity and mobility tops the list” (#313).

## Discussion and Implications

Most of the published literature that explored successful aging from the perspectives of older adults included a small sample size, typically less than 100 participants (e.g., Hilton et al., 2012; Lewis, 2011; Nosraty et al., 2015; Reichstadt et al., 2010; Troutman et al., 2011) and focused on one country. This study is the first to explore exclusivity in older adults across nine countries (Australia, New Zealand, England, Scotland, Wales, Northern Ireland, Ireland, Canada, and the United States). To our knowledge, no previous studies have examined what it means to successfully age multinationally. However, systematic reviews have explored cross-cultural differences (Howell & Peterson, 2020; Reich et al., 2020). For example, Reich and colleagues (2020) reviewed results that scanned 13 countries across seven regions (i.e., North America, Latin America, Scandinavia, Western Europe, the Middle East, Asia, and Oceania), and social engagement and a positive attitude were the most frequently mentioned but independence, physical health, cognitive health, and spirituality were also present (Reich et al., 2020). Howell & Peterson (2020) also had similar findings in their 23 included studies across the Circumpolar North, where autonomy, spirituality, positive attitude, and family support were viewed as essential for successful aging. Results from our study overlap with these in that being actively engaged and independent was the main theme with subthemes of independence and approach to life, which may in part relate to cultural beliefs surrounding independence and to a lesser degree individualism. Additionally, social engagement with family and friends, spirituality, and contributing to the community were also present. Similarly, results from another systematic review that explored layperson perspectives of successful aging (Cosco et al., 2013), factors such as biomedical, external, and psychosocial were identified as three main themes. Compared to biomedical and external factors, many additional themes were identified in psychosocial factors, which supports the importance of deviating from the traditional use of early biomedical successful aging models.

Moreover, research also suggests that those who reported happiness live longer and are mentally and physically healthier (Song et al., 2023), and studies exploring older people’s perspective of successful aging consistently report that having a positive attitude and approach to life is important (Cosco et al., 2013; Howell & Peterson, 2020; Reich et al., 2020). Our results support these findings, but further demarcate meaning in that our theme of resilience and acceptance points not only to one’s outlook on life but also how one needs to develop a sense of acceptance, too. Distilling elements of one’s attitude is useful for research and practice as we seek greater knowledge and understanding with regard to how to successfully age.

Cosco’s and colleagues’ (2013) review also points to participants considering finances, future planning, when to retire, and continuing employment as important factors in successfully aging. These factors are likely to be significant in an aging society where intergenerational living is less likely to occur, and the cost of housing, such as independent living, will be the responsibility of older people. Acknowledging that privilege and accessibility affect financial choices in the present as well as when thinking about the future. Continued employment, for example, may be out of necessity, not choice. Our findings support a paradigm shift of the successful aging discourse that supports self-determination and promotes inclusivity within the individual’s culture and social environment.

### Limitations

There were a few limitations of this study among these were that we relied on a convenience sample of participants recruited primarily through Facebook advertisements. Thus, the sample was predominantly female and high-functioning in activities of daily living (ADLs). We also did not ask for ethnicity or income status. The parameters used on Facebook advertisements included people aged 65 and older and the countries residing in. Given the population that answered this may be indicative of those likely to participate in future surveys using Facebook, researchers are encouraged to specifically target underresearched populations.

Another limitation viewed by some may be the use of a single open-ended survey question rather than what others see as using more robust, traditional qualitative data sources (LaDonna et al., 2018). However, in the current study we allowed participants unlimited space in the text field to define successful aging with the question being prioritized within the survey. Using this data collection method allowed participants across multiple countries to complete the survey at the same time, which is a strength of this study compared to others in successful aging.

Our study sheds light on successful aging from the perspective of older adults across several nations; however, our sample is predominantly Anglo-Saxons with English as their first language, which should contextualize the findings. Additionally, the countries represented in our data have some overlapping cultural beliefs, which certainly influenced the results. Further research endeavors could replicate our study with larger and more diverse samples that could compare other cultures, such as differences in collectivistic cultures as well as data collection from a wider demographic pool. Nonetheless, implications for future research, policy, and practice should seek to delve deeper into the lived experiences of older adults, further refining these themes, which were consistent with previous multinational systematic reviews (Howell & Peterson, 2020; Reich et al., 2020). This may be used to develop scales using quantitative measures to further investigate how person-in-environmental factors, such as gender identity, race and ethnicity, and socioeconomic status, may shape the aging process and influence views on successful aging. Further investigation may also include exploring cultural differences and demographics with unified definitions and reliable comprehensive measures. This, in turn, may be useful for healthcare professionals in assessing and addressing the needs of older adults.

The results from this study demonstrate the multidimensional nature of what it means to be successfully aging according to a multinational sample of English-speaking older adults. These findings complement existing research on successful aging and elevate the voices of older adults’ definition of what it means to age successfully. These results add to the growing body of evidence that suggests successful aging is more than maintaining and improving health, including psychosocial and environmental factors in national and international aging policies (i.e., World Health Organization and United Nations) and discussions about successful aging would assist in promoting the benefits of being engaged, active, independent, financially secure, and “good physical and mental health” for older adults.

## Data Availability

Data and analytic materials will be made available on reasonable request to the authors. This study was not preregistered.
